# Multiplex methylated DNA testing in plasma with high sensitivity and specificity for colorectal cancer screening

**DOI:** 10.1002/cam4.2475

**Published:** 2019-08-12

**Authors:** Guodong Zhao, Hui Li, Zixuan Yang, Zhenzhen Wang, Manqiu Xu, Shangmin Xiong, Shiming Li, XiaoTing Wu, Xiaoyu Liu, Ziwen Wang, Yun Zhu, Yong Ma, Sujuan Fei, Minxue Zheng

**Affiliations:** ^1^ Zhejiang University Kunshan Biotechnology Laboratory Zhejiang University Kunshan Innovation Institute Kunshan Jiangsu China; ^2^ Suzhou VersaBio Technologies Co. Ltd. Kunshan Jiangsu China; ^3^ Department of Gastroenterology Affiliated Hospital of Xuzhou Medical University Xuzhou Jiangsu China; ^4^ Department of Laboratory Medicine Affiliated Hospital of Xuzhou Medical University Xuzhou China; ^5^ Suzhou Institute of Biomedical Engineering and Technology Chinese Academy of Sciences Suzhou Jiangsu China; ^6^ School of Medical Technology Xuzhou Medical University Xuzhou Jiangsu China

**Keywords:** advanced adenomas, colorectal cancer, DNA methylation, multiple biomarkers

## Abstract

Methylated *SEPT9* showed relatively low sensitivity in detecting early stage colorectal cancer (CRC) and advanced adenomas (AA) in plasma. Combination of multiple biomarkers was an effective strategy to improve sensitivity in early stage cancer diagnosis and screening. A new qPCR‐based assay combining the detection of methylated *SEPT9* and *SDC2* (ColoDefense test) was used. Methylation statuses of *SEPT9* and *SDC2* were examined in 40 sets of cancer tissues and paired adjacent tissues, 10 adenomatous polyps and 3 hyperplastic polyps (HP). Then evaluated with 384 plasma samples, including 117 CRC patients, 23 AA patients, 78 small polyps patients, and 166 normal individuals. The limit of detection of ColoDefense was about 25 pg per reaction. Both *SEPT9* and *SDC2* were shown by ColoDefense to be heavily methylated in CRC tissues when compared to paired paracancerous tissues and HP (*P* < .01). The sensitivities for detecting AA and stage I CRC by plasma *SEPT9* methylation alone were 12.1% and 65.0%, and those by plasma *SDC2* methylation alone were 43.5% and 55.0%. In comparison, the sensitivities to detect AA and stage I CRC by ColoDefense improved to 47.8% and 80.0%. The overall sensitivity of ColoDefense in detecting CRC was 88.9% (95% CI: 81.4%‐93.7%) with a specificity of 92.8% (95% CI: 87.4%‐96.0%). Detection of the combinatorial biomarker of methylated *SEPT9* and/or *SDC2* is a powerful, convenient and highly effective strategy for early CRC screening with high sensitivity and specificity.

## Graphical abstract

Colorectal cancer (CRC) is the most common malignancy of gastrointestinal tract and methylated SEPT9 showed relatively low sensitivity in detecting early stage CRC and advanced adenomas (AA) in plasma. We evaluated the performance of the new qPCR‐based assay (ColoDefense test) combining the detection of multiple methylated circulating tumor DNA biomarkers. The ColoDefense test showed high sensitivity and specificity in detecting early stage CRC (stage I) and AA in plasma when compared with methylated SEPT9 alone.

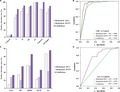

## 1 INTRODUCTION

Colorectal cancer (CRC) is the most common malignancy of gastrointestinal tract and among the three most common cancer types worldwide.^1^ Over 1.8 million new CRC cases and 881 000 deaths are estimated for 2018, accounting for about 1 in 10 cancer cases and deaths.^2^ It is also the fifth most common cancer in China.^3^ And due to the changes toward a more Westernized lifestyle and dietary habits of Chinese population, its incidence has seen steady increase in recent years. During the past decade, the 5‐year relative survival rate of Chinese CRC patients has increased from 47.2% to 56.9%, which, however, was still more than 8% lower than that of the developed countries.^4^, ^5^ Based on epidemiologic studies in developed countries, long‐standing CRC screening and early detection programs had a significant role in reducing morbidity and mortality.^2^

Chinese CRC screening guideline recommends screening adults of 40‐74 years old with fecal occult blood test (FOBT) and followed up with digital rectal exam and colonoscopy.^1^ However, it has been very difficult for the program to reach the entire population, resulting in a very low screening uptake. Due to its invasiveness, bothersome bowel preparation and difficult‐to‐avoid complications, colonoscopy had shown low acceptance rate despite being the gold standard for CRC screening.^6^ Moreover, it is hardly a primary CRC screening method in developing countries with limited resources including China, where FOBT is the most widely used CRC screening method albeit with low accuracy.^7^ Hence, a non‐invasive and more accurate screening method need to be developed to promote early CRC screening.

DNA methylation, the addition of methyl groups to the cytosine residues of DNA, is a form of epigenetic modification that mainly occurs in CpG islands usually present in promoter regions.^8^ Abnormal hypermethylation of certain CpG islands may lead to transcriptional silencing and inactivation of cancer suppressor genes.^9^ In recent years, several studies have reported the application of circulating free DNA biomarkers in CRC diagnosis and screening.^6^, ^10^ The *SEPT9* gene belongs to a class of GTPases involved in numerous cellular processes. It has been shown to have multiple alternatively spliced transcripts encoding at least 5 characterized polypeptides designated v1‐v5, some of which have been associated with ovarian, breast, and other cancers.^11^ The promoter region of the V2 transcript of the *SEPT9* gene has been shown to be hypermethylated and such hypermethylation is specific to CRC carcinogenesis.^12^ Therefore, methylated *SEPT9* became the only blood‐based biomarker approved by FDA for CRC screening, and it has been used clinically for several years.^13^, ^14^ However, the sensitivity of *SEPT9* methylation for CRC detection was relatively low, especially for early stage cancers and advanced adenomas (AA).^4^, ^5^ The *syndecan‐2* protein encoded by *SDC2* gene functions as an integral membrane protein and is known to participate in cell proliferation, cell migration, and cell‐matrix interactions via its receptor for extracellular matrix proteins.^15^ Hypermethylation of *SDC2* has been reported in malignant glioma,^16^ recently, and it was also found to be hypermethylated in the feces or blood samples of most CRC patients.^15^, ^17^ Moreover, *SDC2* methylation showed a higher sensitivity in detecting AA than *SEPT9* methylation.^13^

Combination of multiple biomarkers and/or methods has become a trend in cancer diagnosis and screening to improve sensitivity.^18^ For example, it was reported that the sensitivity for CRC detection was 72.2% and 68.0% respectively, for *SEPT9* methylation and fecal immunochemical test (FIT) individually, and the specificity was 81.5% and 97.4%. When test results for *SEPT9* methylation and FIT were combined, CRC detection rate increased to 88.7% with a specificity of 78.8%.^19^ However, the combination of *SEPT9* and/or *SDC2* methylation for CRC screening has never been reported. In this study, we evaluated the performance of a new blood‐based early CRC screening assay, ColoDefense test, which combined the detection of *SEPT9* and *SDC2* methylation in a single qPCR reaction to improve the detection rate for early stage CRC and AA.

## 2 MATERIALS AND METHODS

### 2.1 Sample collection

Fresh‐frozen CRC cancer tissues (n = 40), paired adjacent paracancerous tissues (n = 40), adenomatous polyps (n = 10), and hyperplastic polyp (HP) (n = 3) were collected at the time of surgery at the Affiliated Hospital of Xuzhou Medical University. The details of age and gender distribution of tissue samples were described in Table 1. All tissue samples were stored at −80°C until use.

**Table 1:** Demographic characteristics of patients examined by ColoDefense test

	Group	Characteristics	Number of patients
Tissue	CRC and adjacent paracancerous tissue (n = 40)	Gender (%)	
		Male	23 (57.5)
		Female	17 (42.5)
		Age	
		Mean (min‐max)	57.4 (33‐78)
		Medium	58
	Adenomatous polyp (n = 10)	Gender (%)	
		Male	7 (70.0)
		Female	3 (30.0)
		Age	
		Mean (min‐max)	56 (24‐80)
		Medium	59
	HP (n = 3)	Gender (%)	
		Male	2 (66.7)
		Female	1 (33.3)
		Age	
		Mean (min‐max)	62 (60‐65)
		Medium	61
Plasma	CRC (n = 117)	Gender (%)	
		Male	64 (54.7)
		Female	53 (45.3)
		Age	
		Mean (min‐max)	61.8 (25‐89)
		Medium	63
	Control (n = 166)	Gender (%)	
		Male	87 (52.4)
		Female	79 (47.6)
		Age	
		Mean (min‐max)	36.6 (21‐69)
		Medium	35
	AA (n = 23)	Gender (%)	
		Male	11 (47.8)
		Female	12 (52.2)
		Age	
		Mean (min‐max)	59.4 (46‐61)
		Medium	60
	SP (n = 78)	Gender (%)	
		Male	52 (66.7)
		Female	26 (33.3)
		Age	
		Mean (min‐max)	56.0 (24‐79)
		Medium	54.5

Abbreviations: AA, advanced adenomas; CRC, colorectal cancer; HP, hyperplastic polyps; SP, small polyps.

Plasma specimens were collected from 117 CRC patients, 23 AA (adenomas with high‐grade dysplasia or with ≥25% villous histologic features or measuring ≥1 cm in the greatest dimension) patients and 78 patients with small polyps (SP, polyps <1 cm or without high‐grade dysplasia or villous component, including 42 non‐advanced adenomas (NAA), 29 HP and seven dysplasia of mild and moderate degrees (MMD). Control plasma specimens were collected from 166 no evidence diseases (normal individuals) and all subjects were verified by colonoscopy at the Affiliated Hospital of Xuzhou Medical University, and the diagnoses of the patients were histologically confirmed by a pathologist (Table 1). Ten milliliter blood was drawn from each subject and stored at 4°C for up to 24 hours. The plasma fractions were then separated and immediately frozen at −80°C until use. The details of tissue and plasma samples were showed in Table 1. This study was approved by the Institutional Review Board of the Affiliated Hospital of Xuzhou Medical University (Ethics Committee reference number: XYFY2018‐KL081), and the informed consent was obtained from all participating patients and healthy control subjects.

### 2.2 DNA extraction, bisulfite treatment, and quantitative real‐time PCR

Genomic DNA was isolated from tissue specimens using a DNeasy Blood & Tissue Kit (Qiagen). For plasma samples, 3.5 mL plasma was extracted using a cfDNA extraction kit (Suzhou VersaBio Technologies Co. Ltd.). Subsequently, bisulfite conversion of purified DNA and purification of the converted product were performed with a bisulfite conversion kit (Suzhou VersaBio Technologies Co. Ltd.). All the kits were used according to the manufacturers’ instructions.

Purified DNA obtained from the above steps was tested by methylation‐specific real‐time qPCR with ColoDefense test (Suzhou VersaBio Technologies Co. Ltd.), a new blood‐based methylation assay for CRC screening. For ColoDefense assay, methylated *SEPT9*, methylated *SDC2*, and an internal control (*ACTB*) can be detected simultaneously in the same multiplex qPCR reaction. Three PCR replicates were performed with purified bisulfite‐converted DNA from each plasma sample, and a single PCR reaction was performed with purified bisulfite‐converted DNA from each tissue sample. qPCR was performed on the LC480‐II thermal cycler (Roche Diagnostics) according to the manufacturers’ instructions.

### 2.3 Analytical performance of ColoDefense test

To evaluate the analytical performance of ColoDefense test, replicate PCR reactions were performed with serially diluted DNA as templates. To examine the limit of detection (LoD) of ColoDefense Test for detecting methylated *SDC2* or *SEPT9* DNA, different amounts of fully methylated genomic DNA were diluted into unmethylated genomic DNA to create mixtures. ColoDefense test were performed in 48 replicates at each DNA concentration for the LoD determination. Each test result was considered “detected” if *ACTB* Cp was less than 32.0, and the Cp values of methylated *SEPT9* and *SDC2* were less than 45.0 and 50.0, respectively.

### 2.4 Data analysis

∆Cp was used to determine the methylation statuses of *SDC2* and *SEPT9* in tissue samples. It was defined as the difference between the Cp values for the target (methylated *SDC2* or methylated *SEPT9*) and the internal control gene (*ACTB*) to normalize for DNA amounts of different samples. The results for plasma specimens were considered “invalid” if the *ACTB* Cp was greater than 35.0, and methylated *SEPT9* and *SDC2* were considered “detected” if their Cp values were less than 45.0 and 50.0, respectively. Methylated *SEPT9* was analyzed using a 1/3 rule in which a plasma sample was scored positive if one of three PCR replicates had a valid amplification curve (1/3 algorithm). And methylated *SDC2* was analyzed using a 2/3 rule, whereby to be called positive, two of three PCR replicates of a plasma sample must have valid amplification curves (2/3 algorithm). The plasma sample would be considered as positive if either methylated *SEPT9* or methylated *SDC2* was positive.

Data were subjected to statistical analysis by IBM SPSS for Windows Version 22.0, and *t* test was used for comparison between two samples at the significance level of *P* < .05. Receiver operating characteristic (ROC) curves were plotted using the mean Cp values from CRC, AA and the Cp values from normal individuals. Because methylated *SEPT9* and methylated *SDC2* were not detected from most normal individuals in the qPCR reaction, we had to set the corresponding Cp values to 50.0 (the maximal number of PCR cycles) for such samples to plot the curve.^14^

## 3 RESULTS

### 3.1 Analytical performance of ColoDefense test

To evaluate the analytical performance of ColoDefense test, mixtures of different ratios of bisulfite‐treated fully methylated and unmethylated genomic DNA were each tested for 48 replicates. As shown in Table 2, methylated *SDC2* qPCR, methylated *SEPT9* qPCR, and ColoDefense test all could detect as low as 12.5 pg fully methylated genomic DNA, equivalent to ~3.8 copies of human genome, and no signal was detected in negative controls. However, the positive detection rate for ColoDefense in 12.5 pg fully methylated genomic DNA was apparently higher than that for methylated *SDC2* (45.8% vs 83.3%) or methylated *SEPT9* (62.5% vs 83.3%) alone. For the analytical sensitivity of the assay, LoD is defined as the target concentration that produces positive result in more than 95% of replicate experiments.^20^ As such, the LoD of methylated *SDC2* qPCR alone, methylated *SEPT9* qPCR alone, and ColoDefense test were 100 pg, 50 pg, and 25pg, respectively, equivalent to ~33.3, ~15.2, and ~7.6 copies of human genome, indicating that the combination of methylated *SDC2* and methylated *SEPT9* could achieve higher sensitivity than either single biomarker alone.

**Table 2:** The analytical performance of ColoDefense test

Fully methylated genomic DNA concentration (pg/reaction)	Methylated *SDC2*		Methylated *SEPT9*		ColoDefense	
	Detected	Detection rate (%)	Detected	Detection rate (%)	Detected	Detection rate (%)
100	48 out of 48	100.0	48 out of 48	100.0	48 out of 48	100.0
50	44 out of 48	91.7	48 out of 48	100.0	48 out of 48	100.0
25	29 out of 48	60.4	40 out of 48	83.3	47 out of 48	97.9
12.5	22 out of 48	45.8	30 out of 48	62.5	40 out of 48	83.3
NC	0 out of 48	0	0 out of 48	0	0 out of 48	0

NC, unmethylated genomic DNA as negative control.

### 3.2 Performance of ColoDefense test on tissue samples

**Figure 1:**
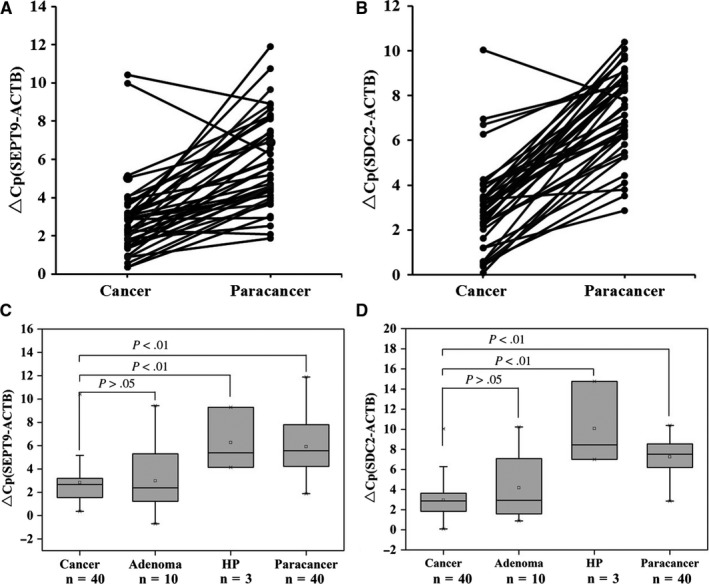
*SEPT9* and *SDC2* methylation in tissue samples. Methylation levels of *SEPT9* (A) and *SDC2* (B) genes in colorectal cancer tissues and paracancerous tissues assessed by ColoDefense test. Methylation levels of *SEPT9* (C) and *SDC2* (D) genes in colorectal cancer tissues compared with adenomatous polyps, hyperplastic polyps, and paracancerous tissues. ∆Cp was defined as the difference between the Cp values for the target (*SEPT9* or *SDC2*) and the internal control gene (*ACTB*)

ColoDefense test was used to quantify methylation levels of *SEPT9* and *SDC2* genes in 40 CRC and paired adjacent paracancerous tissues, 10 adenomatous polyps and 3 HPs. *SEPT9* and *SDC2* methylation levels were higher in 92.5% (37/40) and 97.5% (39/40) of cancer tissues than in their paired adjacent paracancerous tissues (*P* < .01, Figure 1A,B). Compared with HP, *SEPT9* and *SDC2* methylation levels were significantly higher in CRC cancer tissues (*P* < .01, Figure 1C,D) but showed no significant difference in adenomatous polyps when compared with CRC cancer tissues (*P* > .05, Figure 1C,D), thus making ColoDefense test a candidate screening method for CRC and precancerous lesions.

### 3.3 Performance of ColoDefense test in detecting plasma samples

**Figure 2:**
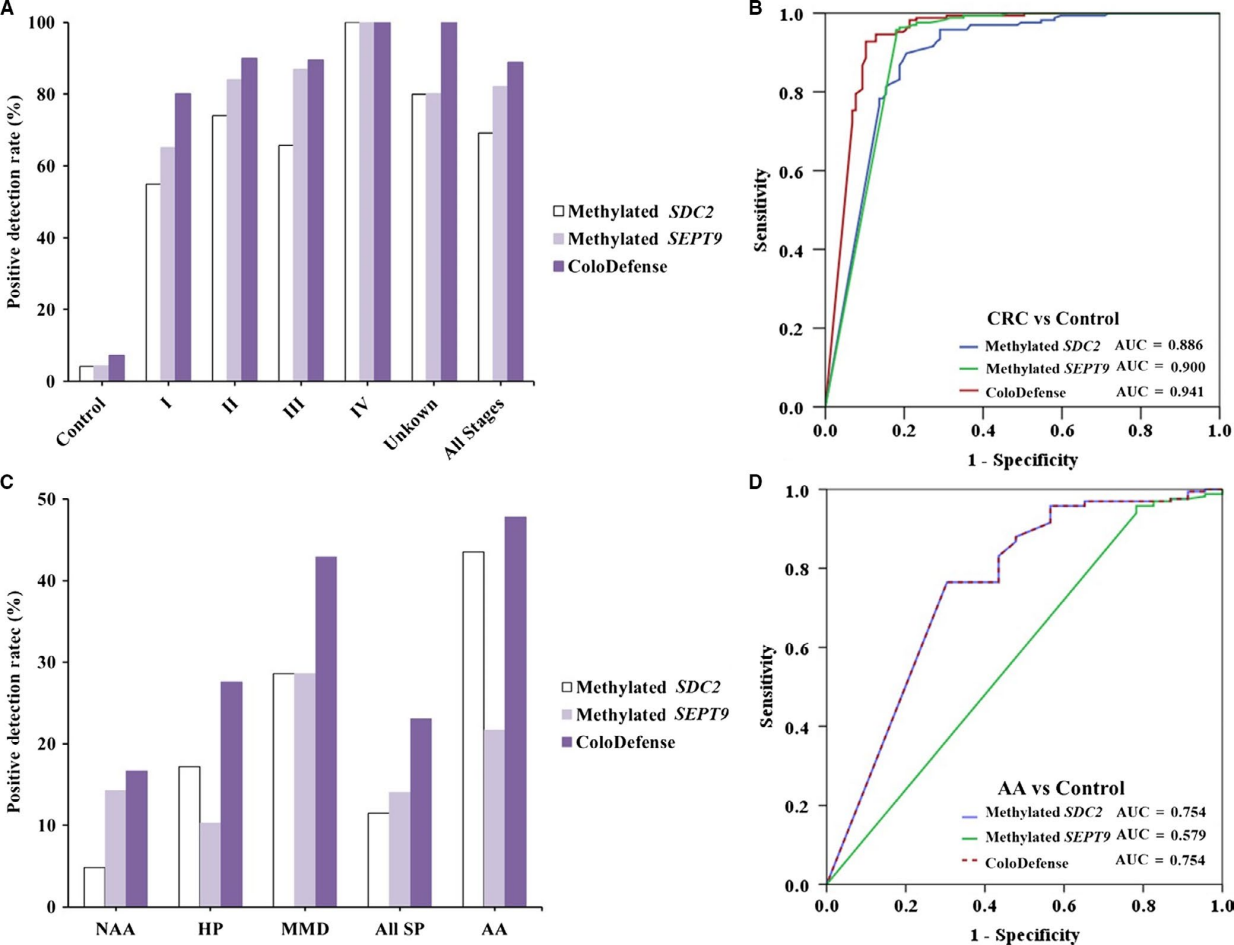
Sensitivity of ColoDefense test in detecting polyps and CRC across stages I‐IV. A, Positive detection rates for control and all stages of CRC. B, ROC curves for ColoDefense test in detecting CRC. C, Positive detection rates for different types of SPs and AA. D, ROC curves for ColoDefense test in detecting AA. AA, advanced adenomas; CRC, colorectal cancer; ROC, receiver operating characteristic; SP, small polyps

To examine the performance of ColoDefense assay for CRC screening, 384 plasma samples were collected from patients of the Affiliated Hospital of Xuzhou Medical University, of which 117 were from CRC patients, 23 from AA patients, 78 from patients with SP, and 166 from normal individuals. The ages of all CRC patients ranged from 25 to 89 with a mean age of 61.8 and a median age of 63. The ages of normal individuals ranged from 21 to 69 with a mean age of 36.6 and a median age of 35 (Table 1). Out of 117 CRC plasma samples whose stages were determined based on the surgically resected specimens, methylated *SEPT9* was detected in 65.0% of stage I (13/20), 84.0% of stage II (42/50), 86.8% of stage III (33/38), 100% of stage IV (4/4), and 80.0% of unknown stage (4/5) samples. Methylated *SDC2* was detected in 55.0% of stage I (11/20), 74.0% of stage II (37/50), 65.8% of stage III (25/38), 100.0% of stage IV (4/4), and 80.0% of unknown stage (4/5) samples. In contrast, with ColoDefense test, the sensitivities improved to 80.0% for stage I (16/20), 90.0% for stage II (45/50), 89.5% for stage III (34/38), 100% for stage IV (4/4), and 100.0% of unknown stage (5/5) CRC (Figure 2A). The sensitivities of methylated *SEPT9* alone, methylated *SDC2* alone, and ColoDefense test for all stage CRC were 82.1% (95% CI: 73.6%‐88.3%), 69.2% (95% CI: 59.9%‐77.3%), and 88.9% (95% CI: 81.4%‐93.7%) with specificities of 95.8% (95% CI: 91.2%‐98.1%), 95.8% (95% CI: 91.2%‐98.1%), and 92.8% (95% CI: 87.4%‐96.0%), respectively. For polyp specimens, the sensitivities of ColoDefense test were 47.8% for AA (11/23), 16.7% for NAA (7/42), 27.6% for HP (8/29), and 42.9% for MMD (3/7) (Figure 2B). Because SP (including NAA, HP and MMD) were always considered benign polyps, the adjusted specificities with SP counted as normal for methylated *SEPT9* alone, methylated *SDC2* alone, and ColoDefense test were 92.6% (95% CI: 88.4%‐95.4%), 93.4% (95% CI: 89.4%‐95.3%), and 87.7% (95% CI: 82.8%‐91.4%), respectively.

Receiver operating characteristic curves for ColoDefense detecting CRC and AA in plasma are shown in Figure 2C,D, respectively. AUC for methylated *SEPT9* alone in detecting CRC was 0.900 (95% CI: 0.857‐0.944) and AUC for methylated *SDC2* alone in detecting CRC was 0.886 (95% CI: 0.843‐0.930). In contrast, ColoDefense test improved the AUC to 0.941 (95% CI: 0.909‐0.973). For detecting AA, AUC for methylated *SEPT9* alone was 0.579 (95% CI: 0.444‐0.715), and AUC for methylated *SDC2* alone was 0.754 (95% CI: 0.635‐0.874), while AUC for ColoDefense test was 0.754 (95% CI: 0.635‐0.874). Overall, the above results demonstrated higher sensitivity of the combinatorial ColoDefense test than either single‐biomarker assay without significant impact on specificity, resulting in better performance of ColoDefense test in distinguishing CRC and AA subjects from normal subjects.

Furthermore, there was no significant difference among the positive detection rates of methylated *SEPT9* alone or methylated *SDC2* alone or ColoDefense test between different ages, genders, or tumor locations (*P* > .05, Table 3). However, the positive detection rates seemed to increase with the increase in tumor sizes. The positive detection rates were 55.6%, 80.5%, and 96.0%, respectively for tumors of <3 cm, 3‐6 cm and >6 cm in sizes by methylated *SEPT9* alone, 33.3%, 71.4%, and 72.0% by methylated *SDC2* alone, and 66.7%, 88.3%, and 96.0% by ColoDefense test. As the positive correlation between larger tumor sizes and higher TNM stages was previously reported,^21^ the apparent positive correlation between positive detection rates and tumor sizes we observed further suggested a positive correlation between positive detection rates and TNM stages.

**Table 3:** Positive detection rates of ColoDefense test for CRC of different ages, genders, tumor locations, and tumor sizes

	Methylated *SDC2* (%)	*P‐*value	Methylated *SEPT9* (%)	*P‐*value	ColoDefense (%)	*P‐*value
Age						
<60 (n = 46)	29 (63.0)	.318	38 (82.6)	.899	41 (89.1)	.947
≥60 (n = 71)	51 (71.8)		58 (81.7)		63 (88.7)	
Gender						
Male (n = 64)	45 (70.3)	.621	55 (85.9)	.229	58 (90.6)	.511
Female (n = 53)	35 (66.0)		41 (77.4)		46 (86.8)	
Location						
Proximal (n = 61)	43 (70.5)	.471	49 (80.3)	.522	53 (86.9)	.537
Distal (n = 53)	34 (64.2)		45 (84.9)		48 (90.6)	
N/A (n = 3)	3 (100.0)		2 (66.7)		3 (100.0)	
Size						
<3 cm (n = 9)	3 (33.3)	.021^a^	5 (55.6)	.088^a^	6 (66.7)	.076^a^
3‐6 cm (n = 77)	55 (71.4)	.956^b^	62 (80.5)	.064^b^	68 (88.3)	.261^b^
>6 cm (n = 25)	18 (72.0)	.041^c^	24 (96.0)	.003^c^	24 (96.0)	.019^c^
N/A (n = 6)	4 (66.7)		5 (83.3)		6 (100.0)	

Abbreviations: CRC, colorectal cancer; N/A, not applicable.

a *P*‐value between <3 cm and 3‐6 cm.

b *P*‐value between 3‐6 cm and >6 cm.

c *P*‐value between <3 cm and >6 cm.

## 4 DISCUSSIONS

Colorectal cancer is one of the most prevalent malignancies globally, and there are various strategies for CRC screening nowadays such as colonoscopy, sigmoidoscopy and FOBT.^22^ As the gold standard for CRC screening, colonoscopy has demonstrated the highest sensitivity and the highest specificity for early detection of colonic malignancies in the average‐risk population. However, its wide acceptance has been limited by some drawbacks, such as post‐polypectomy bleeding and perforation, high cost,^23^ and a significant miss rate of colonic lesions including large abnormalities.^24^

Circulating tumor DNA (ctDNA) predominantly originating from cancer is of great importance to those interested in early cancer detection.^25^ Aberrant DNA methylation, which is considered a hallmark of cancer, can be assessed accurately in ctDNA. Consequently, DNA methylation testing in bodily fluids represents a powerful diagnostic tool in the clinical management of malignant diseases.^17^ Epi proColon 2.0 assay, a blood ctDNA‐based *SEPT9* methylation assay for CRC screening, has recently been approved by both FDA and Chinese FDA.^26^ It showed a sensitivity of 68.2% and a specificity of 78.2% in a large cohort study with 1/3 algorithm.^27^ Another clinical trial reported that its sensitivity for detecting stage I CRC was as low as 35.0%.^28^ And several case‐control studies reported that its sensitivity for detecting CRC ranged from 73.3% to 81.0%.^19^, ^26^ Overall, although methylated *SEPT9* was the only blood based biomarker approved by FDA for CRC screening, its sensitivity did not show significant advantage over FIT.^29^ Meanwhile, most CRC develop from adenomas, among which AA are considered to be the clinically relevant precursors of CRC.^30^ Therefore, screening and intervention for AA has been considered as a primary strategy for prevention and early detection of CRC.^22^ However, the sensitivity of methylated *SEPT9* in detecting AA ranged from 9.8% to 21.6%,^14^, ^27^ indicating the methylated *SEPT9* alone to be hardly a satisfactory screening strategy for colorectal precancerous lesions.

In the present study, a new blood‐based early CRC screening assay, ColoDefense test, was evaluated. It is a combinatorial assay that can detect two methylation biomarkers, *SEPT9* and *SDC2*, simultaneously in a single qPCR reaction. Both *SEPT9* and *SDC2* genes were shown by ColoDefense test to be heavily methylated in CRC tissues when compared to paired paracancerous tissues and HP tissues (*P* < .01). ColoDefense test showed 88.9% sensitivity for detecting CRC and 47.8% for AA detection with a specificity of 92.8% in distinguishing CRC and AA subjects from normal subjects. Compared with Epi proColon 2.0, ColoDefense test showed a significant improvement in sensitivities for detecting CRC and AA, likely because the detection of either methylated *SEPT9* or methylated *SDC2* promoted the LoD of the assay (Table 2) and methylated *SDC2* was a more sensitive biomarker in detecting AA.^6^

Methylated *SDC2* has been reported as a blood‐based biomarker for CRC in recent years. Several studies have reported high sensitivity and specificity of methylated *SDC2* for CRC screening in serum, plasma or stool samples.^10^, ^15^, ^20^ Moreover, sensitivities of methylated *SDC2* from these studies could be improved by nested PCR or normalization of the level of methylated *SDC2* against *ACTB* level. Oh et al showed the LoD of methylated *SDC2* alone without nested PCR was 100 pg genomic DNA, consistent with our result (Table 2). However, the positive detection rate of methylated *SDC2* alone for 10 pg genomic DNA in our study was significantly higher than that in the previous study (45.8% vs 0%).^20^ Moreover, the combination of methylated *SEPT9* or methylated *SDC2* also significantly improved the LoD of ColoDefense test to 25 pg genomic DNA, which was fourfold higher than that of methylated *SDC2* alone and twofold higher than that of methylated *SEPT9* alone.

For early stage (stages I/II) CRC detection, ColoDefense test showed 80.0% and 90.0% sensitivities, significant higher than that of methylated *SDC2* alone (55.0% and 74.0%) or methylated *SEPT9* alone (65.0% and 84.0%). Early stage tumors usually have smaller sizes, and the combination of methylated *SEPT9* or methylated *SDC2* showed a significant improvement in detecting CRC of smaller tumor sizes when compared with either biomarker alone (Table 3). For ColoDefense test, the contribution of methylated *SEPT9* alone in detecting AA appeared to be negligible, and it showed no significant difference among AA, NAA, HP, and MMD (*P* > .05). In contrast, methylated *SDC2* alone showed significant difference between AA and NAA or HP (*P* < .05). Therefore, introducing methylated *SDC2* detection into ColoDefense test made it a powerful tool for the screening of early stage CRC and precancerous lesions. Although the value of AA detection for the early screening of CRC had been addressed in many earlier studies,^31^ most ctDNA‐based CRC screening assays, especially the blood‐based ones, did not show any breakthrough until a multi‐target stool DNA test, Cologuard, was approved by FDA. It could detect 92.3% of CRC and 42.4% of AA with a specificity of 86.7%.^32^ However, its high price and cumbersome procedure^6^ made it unsuitable for developing countries like China. Meanwhile, several stool methylated *SDC2* tests for CRC screening were reported in recently years. Oh et al showed that stool methylated *SDC2* alone had a 90.0% sensitivity for detecting CRC and a 33.3% sensitivity for detecting NAA with a specificity of 90.9%.^20^ Niu et al also reported a stool methylated *SDC2* test that detected 81.1% of CRC and 58.2% of AA with a specificity of 93.3%.^6^ Compared to stool methylated *SDC2* tests, plasma methylated *SDC2* test showed a lower sensitivity for detecting CRC in this study. Such a difference could be due to lower ctDNA level in plasma than tumor DNA in stool and the 2/3 scoring algorithm used by plasma methylated *SDC2* test. On the contrary, the 47.8% and 88.9% sensitivity of the low‐cost ColoDefense test for AA and CRC detection and its 92.8% specificity were quite comparable to those of Cologuard^32^ and stool *SDC2* tests,^6^, ^20^ thus providing a viable alternative for CRC screening and prevention for developing countries.

## 5 CONCLUSION

In this study, we evaluated a new blood‐based early CRC screening assay, ColoDefense test, which detects either of two methylation biomarkers, *SEPT9* and *SDC2*. The results demonstrated that its detection rates for AA and CRC positive were significantly improved upon either methylated *SEPT9* or methylated *SDC2* alone without significant impact on specificity. Therefore, ColoDefense test has the potential to become a powerful, convenient and highly effective screening tool for early CRC screening of high sensitivity and specificity.

## CONFLICTS OF INTEREST

Guodong Zhao and Shangmin Xiong are employees of Suzhou VersaBio Technologies Co. Ltd. Shangmin Xiong is the shareholder of Suzhou VersaBio Technologies Co. Ltd. The other authors declare that they have no competing interests.

## AUTHOR CONTRIBUTIONS

GZ, HL, ZY, and YM performed the statistical analyses and drafted the manuscript. ZY, ZW, MX, SX, SL, XW, XL, ZW, YZ, YM, MZ, and SF participated in sample collection and data analysis. GZ, HL, YM, MZ, and SF conceived of the study and participated in the design and coordination of the study. All authors read and approved the final manuscript.

## ETHICS APPROVAL AND CONSENT TO PARTICIPATE

This study was approved by the Institutional Review Board of the Affiliated Hospital of Xuzhou Medical University (Ethics Committee reference number: XYFY2018‐KL081), and the informed consent was obtained from all participating patients and healthy control subjects, and the study was performed according to the Declaration of Helsinki principles.

## Data Availability

The data sets analyzed during this current study are available from the corresponding author upon request and approval of said request from the respective committees for each cohort.
